# Olfactory epithelium histopathological findings in long-term coronavirus disease 2019 related anosmia

**DOI:** 10.1017/S0022215120002455

**Published:** 2020-11-16

**Authors:** L A Vaira, C Hopkins, A Sandison, A Manca, N Machouchas, D Turilli, J R Lechien, M R Barillari, G Salzano, A Cossu, S Saussez, G De Riu

**Affiliations:** 1 Maxillofacial Surgery Operative Unit, University Hospital of Sassari, Italy; 2 Biomedical Science Department, University of Sassari, Italy; 3 Department of ENT, King's College, London, UK; 4 Department of Histopathology, Charing Cross Hospital and Imperial College Healthcare NHS Trust, London, UK; 5 Histopathology Operative Unit, Department of Medical, Surgical and Experimental Sciences, University of Sassari, Italy; 6 Otorhinolaryngology Operative Unit, University Hospital of Sassari, Italy; 7 Radiology Operative Unit, University Hospital of Sassari, Italy; 8 COVID-19 Task Force of the Young-Otolaryngologists of the International Federations of Oto-rhino-laryngological Societies (‘YO-IFOS’), Belgium; 9 Department of Human and Experimental Oncology, Faculty of Medicine UMONS Research Institute for Health Sciences and Technology, University of Mons (‘UMons’), Belgium; 10 Department of Mental and Physical Health and Preventive Medicine, Luigi Vanvitelli University, Naples, Italy; 11 Maxillofacial Surgery Unit, University Hospital of Naples ‘Federico II’, Italy

**Keywords:** Coronavirus, Smell, Anosmia, SARS-CoV, Olfaction Disorders, Etiology, Pathology

## Abstract

**Background:**

Olfactory dysfunction represents one of the most frequent symptoms of coronavirus disease 2019, affecting about 70 per cent of patients. However, the pathogenesis of the olfactory dysfunction in coronavirus disease 2019 has not yet been elucidated.

**Case report:**

This report presents the radiological and histopathological findings of a patient who presented with anosmia persisting for more than three months after infection with severe acute respiratory syndrome coronavirus-2.

**Conclusion:**

The biopsy demonstrated significant disruption of the olfactory epithelium. This shifts the focus away from invasion of the olfactory bulb and encourages further studies of treatments targeted at the surface epithelium.

## Introduction

Olfactory dysfunction represents one of the most frequent symptoms of coronavirus disease 2019 (Covid-19), affecting about 70 per cent of patients.^1–6^ Many patients recover spontaneously within 15 days; however, severe olfactory dysfunction (i.e. anosmia and severe hyposmia) persists in 7–8 per cent of cases for over two months after clinical onset.^7–9^

The pathogenesis of Covid-19 related olfactory dysfunction has not yet been elucidated.^10^ At the beginning of the pandemic, most authors hypothesised a pathogenesis linked to neuroinvasion of the olfactory bulb, with subsequent neuronal apoptosis.^11,12^ This hypothesis was supported by the neuroinvasive capacity demonstrated by severe acute respiratory syndrome coronavirus-1 in the past,^13^ and by reports of changes in the olfactory bulb on magnetic resonance imaging (MRI) in anosmic patients affected by Covid-19.^14^ However, the hypothesis was refuted by the general tendency for rapid regression of the disorder in many patients and reports that olfactory dysfunction seems to be more common in mild Covid-19 cases.^15,16^

For these reasons, the attention of investigators has shifted to the olfactory epithelium as a possible site of viral damage.^17^ This hypothesis is further supported by: radiological evidence of olfactory cleft oedema in some anosmic patients;^18,19^ proof that the supporting cells of the olfactory epithelium have the highest concentration of viral receptors;^20^ and findings from the first two histopathological reports on animal models^21^ and samples taken from cadavers.^22^

We report the radiological and histopathological findings of a patient who presented with anosmia for more than three months after infection with severe acute respiratory syndrome coronavirus-2 (SARS-CoV-2). To the best of our knowledge, this is the first *in vivo* histopathology report and the first account in a patient with long-lasting anosmia.

## Case report

In early March 2020, a 63-year-old woman presented with mild fever, with intense asthenia, anosmia and hypogeusia. The patient had no significant co-morbidities or previous olfactory or gustatory disturbances. In 5 days, fever and asthenia completely regressed, while chemosensory disturbances remained unchanged. At the end of March, after the detection of several cases of Covid-19 at the patient's workplace, she was subjected to a nasopharyngeal swab, which was negative, and a serological test, positive for SARS-CoV-2 immunoglobulin (Ig) IgG and IgM.

In June, given the persistence of anosmia for over three months, the patient was admitted to the maxillofacial surgery department of the University Hospital of Sassari for diagnostic investigations.

At the time of admission, nasopharyngeal swab and serological tests were repeated, which revealed positivity only for SARS-CoV-2 IgG. In the previous three months, the patient had not taken any specific medication. The olfactory and gustatory functions were objectively evaluated with psychophysical tests as per our protocol,^2,23,24^ which detected anosmia and severe hypogeusia.

**Fig. 1.**
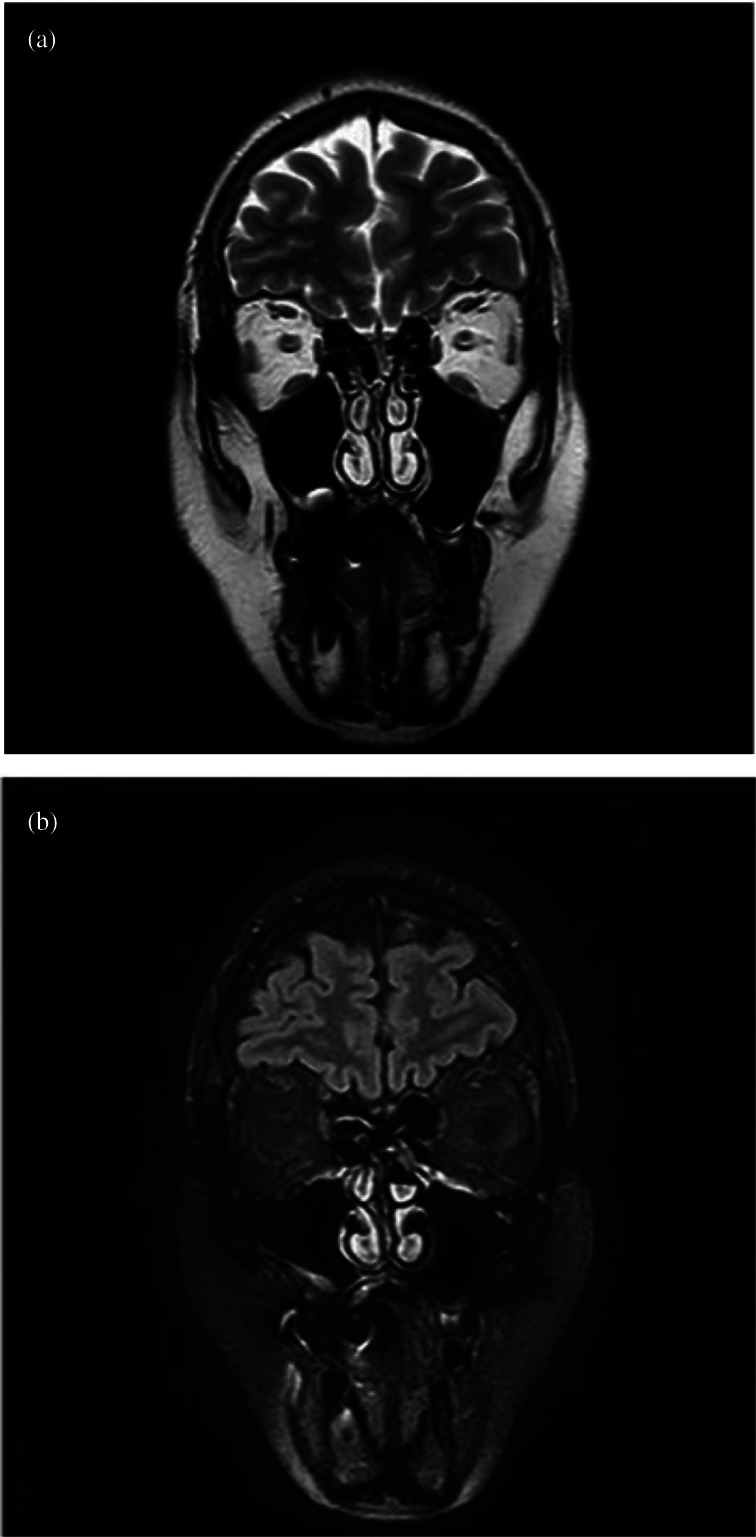
Magnetic resonance imaging did not reveal any pathological findings: the olfactory bulb and clefts were of normal volume, without signal anomalies. Coronal scans of: (a) T2-weighted fast spin echo sequence, and (b) T2-weighted fluid-attended inversion recovery with fat suppression sequence.

The patient was first subjected to contrast-enhanced MRI of the nasal cavities and brain. The examination did not reveal any pathological findings: the olfactory bulb and clefts were of normal volume, without signal anomalies (Figure 1).

After providing signed written consent and being informed about the risks of the procedure, the patient underwent a biopsy of the left olfactory epithelium. The endoscopic procedure was conducted under general anaesthesia, as previously described by other authors.^25^ During the procedure, a swab was performed directly on the olfactory epithelium, which showed as negative for residual Covid-19.

### Histopathological findings

**Fig. 2.**
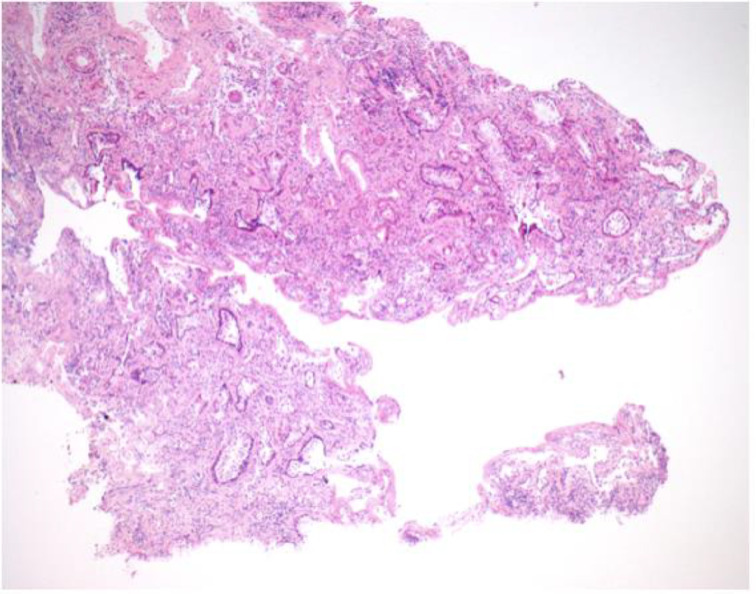
Low power stain shows mucosa devoid of surface epithelium. There is mild chronic inflammation, but no evidence of acute inflammation. (H&E; ×25)

**Fig. 3.**
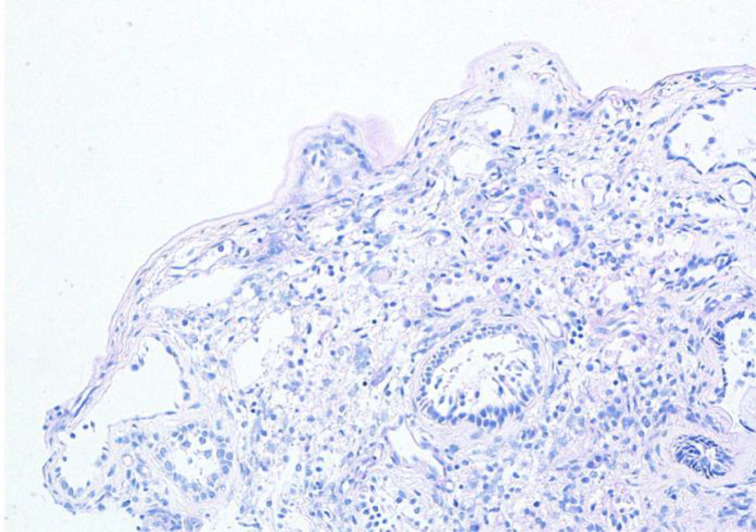
Special stain does not highlight surface basement membrane or inflammatory exudate. (Periodic acid–Schiff; ×100)

The mucosal biopsy sections measured 8 mm × 4 mm in maximum dimension. There was extensive loss of surface epithelium (Figure 2), with no associated surface fibrin or inflammatory exudate (Figure 3). The architecture of glands in the lamina propria was maintained. A minimal chronic lymphocytic inflammatory infiltrate was present (Figure 2). No eosinophils or mast cells were identified.

**Fig. 4.**
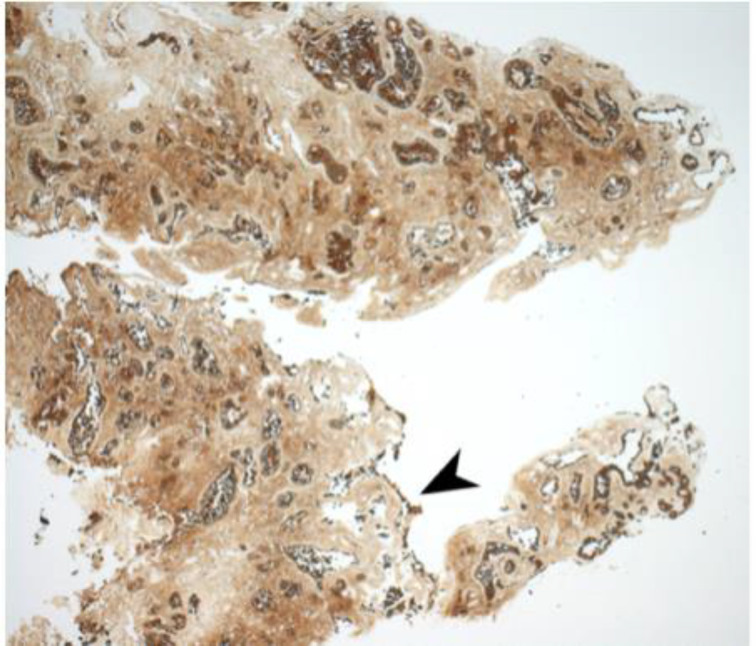
Immunostain showed possible attenuated residual surface epithelial cells, stained brown (arrowhead). (Pan-cytokeratin immunostain; ×25)

**Fig. 5.**
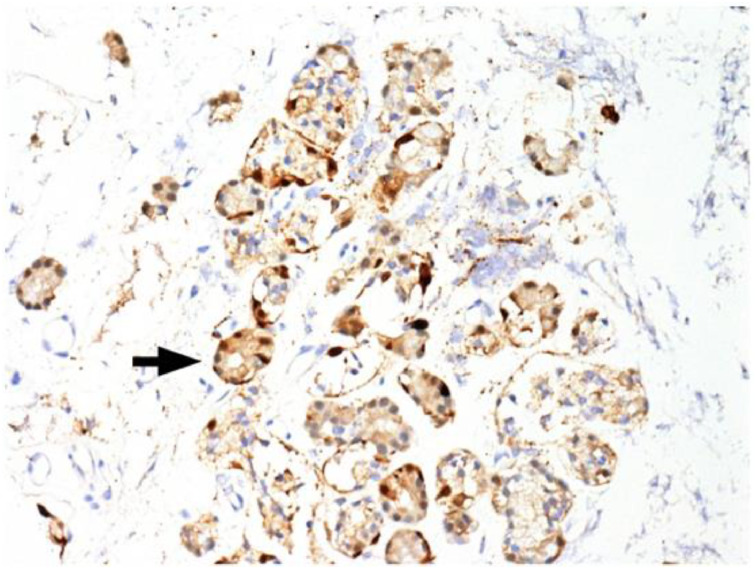
Immunostain shows strong nuclear and cytoplasmic positivity in scattered cells in structures compatible with Bowman's glands (arrow). The same immunostain highlighted small nerve bundles, possibly of trigeminal origin, not illustrated in this field. (S100 immunostain; ×200)

**Fig. 6.**
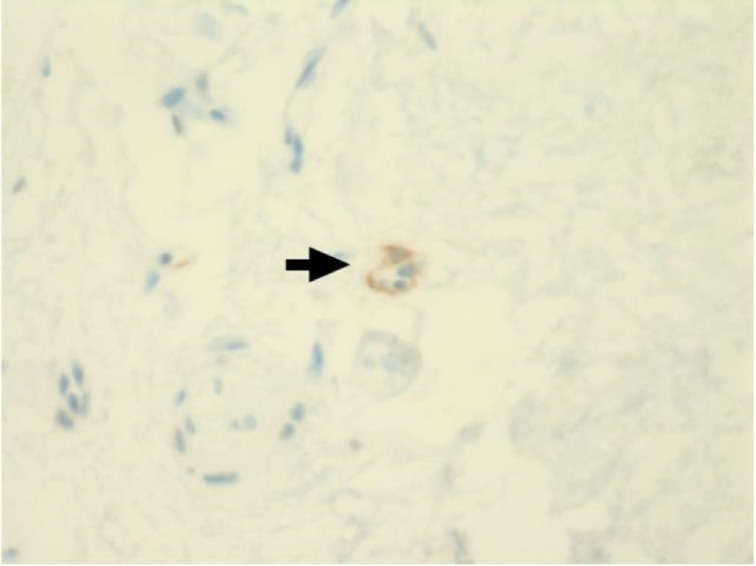
Immunostaining for angiotensin-converting enzyme 2 (ACE2) receptor showed focal membrane staining in cells that were also positive for S100 in Bowman's glands (arrow). (ACE2 immunostain; ×200)

**Fig. 7.**
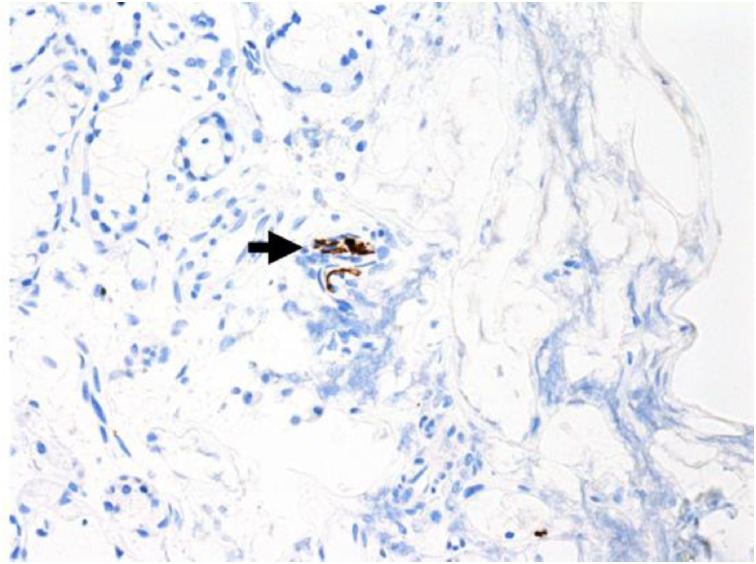
Focal positive staining for neurofilament immunostain highlighted small neurites and nerve bundles in lamina propria (arrow). (Neurofilament immunostain; ×100)

Immunohistochemical staining for pan-cytokeratin AE1/AE3 antibodies demonstrated only very focal residual attenuated surface epithelium (Figure 4). There was strong nuclear and cytoplasmic positivity for S100 immunostain in scattered cells within structures, compatible with Bowman's glands (Figure 5); the same immunostain highlighted small nerve bundles, possibly of trigeminal origin. Immunostaining for angiotensin-converting enzyme 2 (ACE2) receptor showed focal membrane staining in the S100 positive cells in Bowman's glands (Figure 6). There was focal positive staining for synaptophysin, and neurofilament immunostain highlighted small neurites and nerve bundles in lamina propria (Figure 7). No abnormal neural proliferation was identified.

Based on the results of the histopathological examination, the patient began systemic cortisone therapy with prednisone, starting with 75 mg/day and tapering the dose for 15 days. The patient received nasal irrigation with betamethasone, ambroxol, and Rinazina® for 30 days. At the end of therapy, the patient reported a slight improvement in chemosensory symptoms. Psychophysical tests revealed severe hyposmia and moderate hypogeusia. A new cycle of cortisone therapy was scheduled for September.

## Discussion

Nasal congestion associated with viral infections of the upper respiratory tract often causes transient anosmia.^26^ However, the olfactory dysfunction in Covid-19 is not characteristically associated with rhinitis symptoms; the cause is therefore more likely to be due to injury to the olfactory epithelium or olfactory apparatus than secondary to nasal obstruction. The exact location of this damage remains uncertain given the paucity in the literature of histopathological studies on samples taken from Covid-19 patients.

In the case reported here, in which anosmia persisted for three months after Covid-19 infection, the MRI findings excluded any macroscopic inflammation affecting the olfactory bulb, the pathway or the olfactory epithelium (Figure 1). These findings are consistent with those of Galougahi *et al*.,^27^ but are in contrast with reports from other authors, who detected olfactory cleft inflammation^18,19^ or an increase in olfactory bulb volume^14^ in the early stages of anosmia. Our radiological findings suggest that an aetiology linked to olfactory bulb impairment is unlikely. Only a bulbar biopsy, clearly impossible in patients recovered from Covid-19, could rule out a macroscopically non-evident nerve injury.

Kirschenbaum *et al*.^22^ reported on the post-mortem histological analysis of olfactory epithelium in two elderly male patients who died 6 and 8 days after hospital admission. They demonstrated findings consistent with an inflammatory neuropathy, with prominent leukocytic infiltrates in the lamina propria, focal atrophy of the mucosa, and digestion chambers in the olfactory nerve fibres suggestive of axonal damage. Both brains showed perivascular leukocytic infiltrates, predominantly in the basal ganglia and intravascular microthrombi.

Using a mouse model to study the effects of SARS-CoV-2, Bryche *et al*.^21^ demonstrated extensive olfactory epithelium damage within days of inoculation, almost exposing the olfactory sensory neurones. The virus was shown to be present in the olfactory epithelium at day 2, but was already decreasing by day 4. The virus was not demonstrated in the olfactory bulb or cortex. In keeping with the work of Brann *et al*.,^28^ Bryche *et al*.^21^ demonstrated infection of the supporting sustentacular cells, but not the olfactory neurons themselves. Desquamation, however, affected both infected and non-infected cells, with the olfactory neurons showing loss of cilia.

The ACE2 receptor is considered the portal of entry for SARS-CoV-2, and upregulation of ACE2 receptors may increase the risk of infection.^29^ No upregulation was detected in our patient's biopsy.

Previous studies in patients with non-coronavirus post-viral olfactory loss show long-lasting changes in the olfactory epithelium. Yamagishi *et al*.^30^ showed thinning of the epithelium with loss of the characteristic three-layer structure; there was also a reduction in the number of olfactory receptor cells, while those that were present lacked cilia. Patients with hyposmia demonstrated more ciliated olfactory neurons. In anosmic patients, olfactory vesicles were absent; in hyposmic patients, they were reduced in number.^31^ Jafek *et al*.^32^ demonstrated patchy regeneration of the olfactory epithelium interspersed with respiratory epithelium, and in some cases the olfactory epithelium was replaced by metaplastic squamous epithelium.

These reports are all consistent with our results. The findings suggest that disruption and desquamation of the olfactory epithelium is the underlying mechanism in Covid-19 related olfactory dysfunction. Failure of epithelial repair leads to thinning and loss of the olfactory dendrites. Patchy recovery may lead to hyposmia and/or dysosmia.

• A patient presented with anosmia for more than three months after severe acute respiratory syndrome coronavirus-2 infection
• The patient was first subjected to contrast-enhanced magnetic resonance imaging of the nasal cavities and brain
• The examination did not reveal any pathological findings: the olfactory bulb and clefts were of normal volume, without signal anomalies
• A biopsy, taken three months after onset of coronavirus disease 2019 related anosmia, demonstrated massive olfactory epithelium disruption
• These findings shift the focus away from olfactory bulb invasion and towards treatments targeted at surface epithelium

The findings have important implications when considering novel treatment options that could be targeted to the olfactory epithelium. There is evidence to support steroid rinses, but not intranasal steroid sprays;^33^ this may reflect the greater ability of steroid rinses to reach the olfactory epithelium. In a small pilot study,^34^ submucosal injection of platelet rich plasma into the olfactory epithelium in seven patients with hyposmia was associated with significant improvement, but no benefit was found in two anosmic patients. There was no control arm in that study, but further study is certainly warranted. A number of other topical agents have been investigated in small studies, but in a recent evidence-based review none were considered to provide sufficient evidence about which to make any treatment recommendations.^35^ Given the large numbers of patients affected, this must be made a research priority.

## Conclusion

The biopsy, taken three months after the onset of Covid-19 anosmia, demonstrated massive disruption of the olfactory epithelium. This shifts the focus away from olfactory bulb invasion and encourages further studies of treatments targeted at the surface epithelium.
